# Bio-Based Poly(butylene succinate)/Microcrystalline Cellulose/Nanofibrillated Cellulose-Based Sustainable Polymer Composites: Thermo-Mechanical and Biodegradation Studies

**DOI:** 10.3390/polym12071472

**Published:** 2020-06-30

**Authors:** Oskars Platnieks, Sergejs Gaidukovs, Anda Barkane, Aleksandrs Sereda, Gerda Gaidukova, Liga Grase, Vijay Kumar Thakur, Inese Filipova, Velta Fridrihsone, Marite Skute, Marianna Laka

**Affiliations:** 1 Institute of Polymer Materials, Faculty of Materials Science and Applied Chemistry, Riga Technical University, P.Valdena 3/7, LV, 1048 Riga, Latvia; oplatnieks@gmail.com (O.P.); barkaneanda@gmail.com (A.B.); aleksandrs.sereda@rtu.lv (A.S.); 2 Institute of Applied Chemistry, Faculty of Materials Science and Applied Chemistry, Riga Technical University, P.Valdena 3/7, LV, 1048 Riga, Latvia; Gerda.Gaidukova@rtu.lv; 3 Institute of Silicate Materials, Faculty of Materials Science and Applied Chemistry, Riga Technical University, P.Valdena 3/7, LV, 1048 Riga, Latvia; grase.liga@gmail.com; 4 Biorefining and Advanced Materials Research Center, Scotland’s Rural College (SRUC), Kings Buildings, Edinburgh EH9 3JG, UK; Vijay.thakur@sruc.ac.uk; 5 Latvian State Institute of Wood Chemistry, LV, 1006 Riga, Latvia; inese.filipova@inbox.lv (I.F.); fridrihsone.velta@inbox.lv (V.F.); polarlapsa@inbox.lv (M.S.); lamar@edi.lv (M.L.)

**Keywords:** biopolymer, sustainable composites, thermo-mechanical properties, melt processing, wood plastic composite, biodegradability

## Abstract

Biodegradable polymer composites from renewable resources are the next-generation of wood-like materials and are crucial for the development of various industries to meet sustainability goals. Functional applications like packaging, medicine, automotive, construction and sustainable housing are just some that would greatly benefit. Some of the existing industries, like wood plastic composites, already encompass given examples but are dominated by fossil-based polymers that are unsustainable. Thus, there is a background to bring a new perspective approach for the combination of microcrystalline cellulose (MCC) and nanofibrillated cellulose (NFC) fillers in bio-based poly (butylene succinate) matrix (PBS). MCC, NFC and MCC/NFC filler total loading at 40 wt % was used to obtain more insights for wood-like composite applications. The ability to tailor the biodegradable characteristics and the mechanical properties of PBS composites is indispensable for extended applications. Five compositions have been prepared with MCC and NFC fillers using melt blending approach. Young’s modulus in tensile test mode and storage modulus at 20 °C in thermo-mechanical analysis have increased about two-fold. Thermal degradation temperature was increased by approximately 60 °C compared to MCC and NFC. Additionally, to estimate the compatibility of the components and morphology of the composite’s SEM analysis was performed for fractured surfaces. The contact angle measurements testified the developed matrix interphase. Differential scanning calorimetry evidenced the trans-crystallization of the polymer after filler incorporation; the crystallization temperature shifted to the higher temperature region. The MCC has a stronger effect on the crystallinity degree than NFC filler. PBS disintegrated under composting conditions in a period of 75 days. The NFC/MCC addition facilitated the specimens’ decomposition rate up to 60 days

## 1. Introduction

Global plastic production has seen a steady increase of 10–20 million tons each year with production volumes above 350 million tons a year, while the industry remains mainly oil-based [1,2]. This has caused many concerns over long term sustainability and waste management. More than 50% of the generated plastic waste is discarded in land fields leaving permanent environmental damage and reducing useable land area [2,3]. As a results of this, a move to a bio-based and circular economy has started to achieve full life cycle management [4,5]. Packaging industry with short life spawn products is the biggest contributor to plastic pollution and has dominant use of primary plastics [3,6,7]. Different bio-renewable resources such as biomasses, microbial sources and agro can be used to derive a new class of sustainable polymers as these polymers do not release different toxic elements through the process of their degradation, which labels them as eco-friendly materials [8]. Poly (butylene succinate) (PBS) and poly (lactic acid) (PLA) are a new class of polyesters that have been named as one of the most promising replacements for conventional oil-based polymers and have also been classified as biodegradable [9,10,11]. Indeed, bio-based plastics have shown a great potential for functional and structural applications in agriculture, biomedical engineering and equipment, packaging and water treatment systems [12]. Thus, the advancement of ecological and sustainable materials involves numerous industrial sectors, but next-generation material development requires a sustainable reinforcement selection [13]. Bio-based fillers can be obtained as residues from various industries not only offering economically viable solutions but also being biodegradable, which can significantly reduce recycling costs of the composite materials [14]. While new social practices and technological infrastructure is needed, the befits are clear for sustainable material application and the markets have been growing substantially.

As we tackle some of the biggest challenges like biodegradable packaging, the material properties must be suitable, but PLA while being known as a versatile polyester has a major weakness related to its brittleness [15]. Thus, PBS has been emerging as an alternative with great mechanical properties, ductility and better thermal properties. It has similar processability and properties to widely used polyolefins polyethene and polypropylene. It is synthesized via polycondensation process from 1,4-butanediol and succinic acid and can be processed from either petroleum-based chemicals or biobased ones, but currently, only a succinic acid portion comes from biomass [6,10,16].

Despite PBS being a promising material, it has some drawbacks at the certain application that require high tensile strength and elastic modulus [17,18]. To further increase PBS mechanical properties and thermal resistance extensive studies have been done with various fillers [1,10]. High versatility of the PBS matrix has been proven in various advanced applications like tissue engineering [19], electrospinning [20] good electrical conductivity with carbon-based fillers [21] and even light weight foams have been prepared [22]. Bio-based fillers are often preferred over mineral-inorganic types because they have advantages like renewability, sustainability, abundance, biodegradability, low density [6,18]. It could be also said that they further comply with the bio-based and biodegradable concept design of sustainable materials and allow usage of recycled cellulose or other bio-polymers for circular economy [23]. Several studies have demonstrated effectiveness of combining bio-based polyesters with bio-based fillers like poly(butylene adipate-co-terephthalate) (PBAT) with thermoplastic starch for active packaging [24], PBAT/PLA blends with Babassu for mulch films [25], chitosan blends with various bio-polyesters like PBS, PLA and others demonstrated antimicrobial activity useful for food preservation and packaging [26].

Lignocellulosic materials have several advantages over other bio-based fillers that make them suitable for their usage as reinforcement for an appropriate polymer matrix [27,28]. The abundance of cellulose in nature means there is plenty of raw material for the ever-growing need for biocompatible and eco- friendly products [29,30]. Various forms and treatment methods have been developed to process cellulose resulting in classifications like microcrystalline cellulose (MCC), nanocrystalline cellulose (NCC) and nanofibrillated cellulose (NFC). Growth in research paper publications devoted to this topic is exponential in the last two decades. More than 500 papers are published each year with such keywords [8,31]. Fibrillated nanocellulose is regarded as mechanically stronger reinforcement due to large aspect ratio, increased surface area, which yields much stronger hydrogen bonding but this comes with drawbacks like complicated and expensive production and most importantly challenging dispersion in polymer materials, which often require expensive solvents that are not suitable for industrial application [32]. Thus, wet processing is usually applied for NFC polymer blends that involve water soluble polymers that can preserve structure after drying [33]. It is often suggested that similar mechanism of polymer coating should be used for freeze-drying process [34], but it has been reported that freeze-drying in aerogels can preserve NFC structure [35]. Crystalline cellulose preserves strongest structural parts of cellulose due to removal of amorphous phase and usually results in good particle size distribution, while ability to prepare powder enables applications in melt blending process [31]. While cellulose treatment methods create unique structures, the synergy has not been widely studied and comparisons are indirect.

MCC is the first filler that authors have selected for this research. Conventional preparation of MCC involves alpha-cellulose treatment with an extreme quantity of the mineral acid [36], but thermocatalytic destruction method has been developed and further advanced to obtain fine powder [37] which is the type of MCC used in this research. MCC presents an exclusive capability to advance the thermal, morphological, and mechanical properties of the polymer composites [38,39]. Some reports suggest that MCC possess Young’s modulus of 86–163 GPa and MCC filler has the high potential to considerably strengthen polymers at various loadings of the fillers [40]. NFC was selected as the second filler. High-pressure homogenization of a suspension of cellulose is carried out to prepare NFC in water or other mechanical treatments [7,32]. This yields structure with substantial benefits over those of micro-sized fillers such as higher aspect ratio and higher specific area and better properties as a reinforcing phase in the composites [30,36]. However, there are some drawbacks in the usage of NFC and they are related to the increased costs in the preparation and challenging cellulose transfer from suspensions to polymer melt [30,41].

To the best of our knowledge, previous researches carried out with PBS composites focuses more on various natural fibers [42] and bio-based residues like agro-flour [43] and modified cellulose [44] or PBS matrix grafting [45] or other types of modifications like the addition of PLA [46]. These researches have indicated enhanced mechanical characteristics such as tensile strength and modulus and an increase in storage modulus during the dynamical mechanical analysis. The biodegradation aspect of PBS composites indicates that bio-based fillers increase disintegration speed [42]. Research suggests that nanocellulose incorporation into PBS matrix can increase barrier properties which makes such composites more appealing for packaging industry [10]. PBS has very high thermal stability, which enhances cellulose based fillers but the thermal degradation temperature for the composites decreases from around 400 °C to 350 °C [47]. It is worth mentioning that the crystallinity of the polymer material after filler incorporation is usually reduced resulting in more amorphous polymer matrix structure. It has been reported that addition of 5 wt % NFC increased tensile strength by 23% for polyvinyl alcohol [48] and 21% for PLA [49]. Addition of modified cellulose-acetate nanofibers to PBS resulted in tensile strength being enhanced from 13% up to 52% with varied concentrations from 2 up to 15 wt % as well as using random and aligned fibers [44]. Therefore, the use of cellulose as reinforcement for PBS has huge potential in the development of ecology friendly functional composites.

We take this opportunity to research the synergy of micro and nano cellulose incorporation into PBS matrix using the industry-standard approach with the melt blending process. Not much research is done when both micro and nanocellulose fillers are used at the same time outlining the lack of direct comparison of the unique advantages of each filler. We have selected preparation methods with similar energy consumption and labour amount to reduce environmental impact and select best industrial grade filler. Two single filler compositions of NFC and MCC were prepared and studied together with three NFC/MCC compositions and reference pristine PBS sample. We aimed to compare application of the NFC and MCC fillers at 40% total loading by modifying NFC/MCC ratio from 7/3, 5/5 and 3/7. Key characteristics of the for all the prepared composites have been studied including thermal, mechanical, thermo-mechanical, surface properties and degradation in composting conditions.

## 2. Materials and Methods

### 2.1. Materials

BioPBS™ FZ71PB^®^ polybutylene succinate produced by PTT MCC Biochem Company Ltd. (Thailand) was purchased in the form of pellets. According to the description from the manufacturer, this PBS grade is designed for industrial applications like extrusion, injection moulding and paper coating. The properties are density 1.26 g/cm^3^, melt flow rate (190 °C, 2.16 kg) 22 g/10 min, and melting temperature 115 °C.

**Figure 1:**
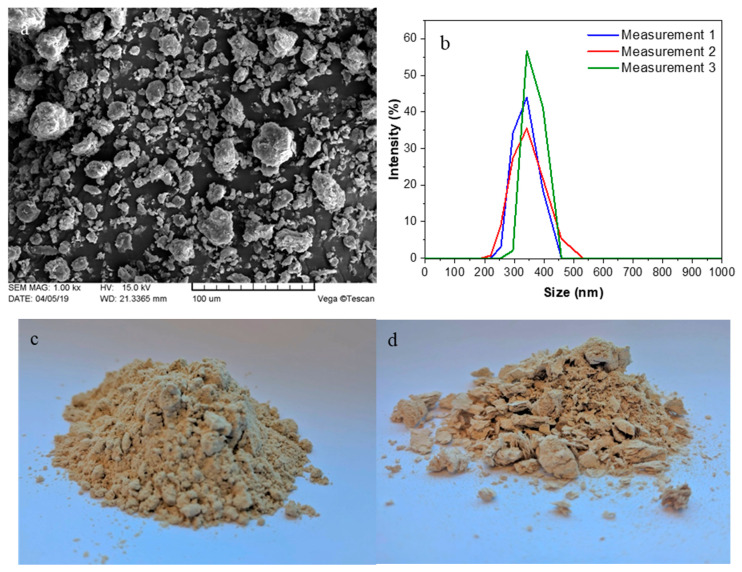
(**a**) Scanning electron microscopy micrograph of microcrystalline cellulose (MCC); (**b**) dynamic light scattering analysis of nanofibrillated cellulose (NFC); (**c**) dry MCC filler and (**d**) freeze-dried NFC filler.

Microcrystalline cellulose was prepared from bleached wood pulp using a method reported by the authors [50]. This method consists of wood pulp treatment with hydrochloric acid followed by filtration and washing with distilled water, drying and dry powder milling of the cellulose for 15 h. The SEM image shows MCC particles with a size from 20 to 40 μm (Figure 1a).

Nanofibrillated cellulose gel-like dispersion was prepared using mechanical treatment with a high shear force following the previously established method [51]. NFC fabrication is similar to MCC preparation but involves addition of water after the drying process and wet milling of the cellulose for 15 h. The size of NFC particles was around 300 nm, as evidenced by dynamic light scattering measurements (Figure 1b). Freeze drying with liquid nitrogen was performed to obtain dry NFC powder (Figure 1d).

### 2.2. Preparation of Composites

PBS was dried in a vacuum for 5 h in 80 °C according to manufacturer’s recommendations. MCC and NFC in the form of powders were dried in a vacuum for 24 h in 60 °C. The composites were prepared with Brabender^®^ Mixer 50EHT (Germany) with the blending temperature set to 140 °C and the rotation speed to 70 rpm. The pure PBS sample was processed; 5 composites were prepared with 40 wt % of MCC, NFC and MCC/NFC combinations in PBS matrix. The prepared compositions are summarized below in Table 1.

### 2.3. Processing of Composites

Carver CH 4386 hydraulic press (USA) was used to prepare thin films having a thickness of 0.1 and 0.3 mm for tests. Mini-Jector #55-1E injection molding device (USA) was used to prepare dumbbells shaped samples for tensile tests. Compression molding was performed at a temperature of 140 °C for 5 min and 3 metric ton pressure, followed by rapid cooling to room temperature between thick steel plates. An injection molding device was set at the temperature of 190 °C and samples were cast in the steel molds.

### 2.4. Characterization

Thermogravimetric analyses (TGA, Mettler, USA) were used to assess sample thermal degradation on a TGA1/SF analyzer from Mettler Toledo for about 10 mg samples with a heating rate of 10 °C min^−1^ in the temperature range from +50 °C up to +600 °C under an air atmosphere.

Further thermal testing was performed on DSC-1 from Mettler Toledo (USA) and calorimetric properties were measured under a nitrogen atmosphere for samples having an average weight of 10 mg. The protocol used for differential scanning calorimetry was heating, and cooling followed by second heating in the temperature range from 25 to 150 °C with heating/cooling rate of 10 °C min^−1^ and samples were kept for 5 min at 150 °C and 25 °C to obtain more precise results.

The degree of the crystallinity for pure polymer and composites can be calculated using the following equation

χ = ΔH_c_/ΔH_m_^0^(1 − W_f_) × 100%, (1)

where ΔH_c_ corresponds to the measured crystallization enthalpy of the specimen, ΔH_m_^0^ is the theoretical melting enthalpy of 100% crystalline polymer (200 J/g for PBS [6]) and W_f_ is the cellulose fiber weight content in the composition.

The prepared composites specimens were conditioned overnight at temperature of 20 °C and the relative humidity of 50%; the dimensions were measured separately before testing. A Tinius Olsen 25ST testing machine (USA) was used to measure the tensile strength using a minimum of 5 samples for each composition with at a 0.2 mm/min constant crosshead speed.

Storage modulus, loss modulus and damping factor of the composites were characterized using Mettler SDTA861e dynamic mechanical analyzer (USA). Experiments were conducted in the tension mode with frequency 1 Hz, elongation of 10 μm, force of 10 N, heating rate of 3 °C min^−1^ and in temperature ranging from −70 to +70 °C. The dimensions of the samples tested were 8.5 × 4 × 0.3 mm.

The composites fractured surfaces were examined using an SEM Hitachi Tabletop Microscope TM3000 (Japan). The composite specimens were fractured using liquid nitrogen and used as they were, to obtain images in different magnifications with a voltage of 15 kV. For MCC powder sample TS Vega Tesca5136M (Czech Republic) was used with a voltage of 15 kV.

The disintegration degree of the obtained composite material was studied under aerobic simulated composting conditions at 58 °C and water content at 50 wt %. Thin films were cut into the sample shapes (25 mm × 25 mm × 0.10 mm) and 5 samples for each composition were sandwiched between sieves. Specimens were submerged at a depth of 5 cm in a commercial compost soil that consist of local Latvian swamp peat (Formoss, Latvia) and packed in plastic containers. The range of pH values from 5.7 to 6.3 was obtained from the measurements. The laboratory-scale test was used to determine disintegration and before the measurements, specimens were vacuum dried for 2 h at 60 °C. The specimen’s weight was measured before the test (reference abbreviated as 0 days) and at following days 10, 20, 30, 40, 50, 55, 60, 65, 70, 75. Average mass loss in percentage was calculated after measurements.

The contact angle of the pristine PBS and composite specimens was analyzed with a Theta Lite optical tensiometer (Attension^®^, China) using static sessile drop method. Five separate measurements with drops (2 μL) of each test liquid were deposited on the surface of the specimen and the measurements were obtained in 30 s. Distilled water, glycerol and diiodomethane were selected as liquids and measurements were conducted at 20 °C. Total surface free energy (SFE) and its dispersive, polar components were calculated from contact angle values using Attension^®^ original software via Owen, Wendt, Rabel and Kaelble method (OWRK). The Zisman model was used to calculate critical SFE values.

## 3. Results and Discussion

### 3.1. Materials and Energy Flows

Table 2 presents the materials and energy flows for the manufacture of the two types of celluloses fillers—MCC and NFC at the laboratory scale for method that is optimized for around 50 g production but for consistency is recalculated for 1 kg of filler production. The raw-material processing steps are very similar for kraft cellulose and require treatment with diluted hydrochloric acid 20 L, filtering and washing with distilled water which is than dried for 8 h in thermostat, resulting in 224 kWh energy consumption and dry cellulose. Before milling the cellulose for NFC preparation was suspended in water while the cellulose for MCC preparation was milled dry. Both processes require 15 h of jar milling that require 210 kWh electric energy. After milling, MCC was ready for the composite preparation in solid powder state, while NFC was obtained as gel and required further freeze-drying to obtain solid filler for melt processing. The freeze-drying process requires 417.6 kWh, which results in total energy 851.6 kWh for NFC preparation compared to 434.0 kWh for MCC preparation a difference of almost two-folds. Emissions were evaluated and both processes resulted in very similar amounts that consisted of 0.009 kg of solid waste mainly attached to devices and lost in cleaning and small amount of dust 0.001 kg, additionally around 20 L of acidic water is produced.

As seen in Table 2 the energy required in production for both MCC and NFC are equal and material input demand slightly more distilled water for NFC production. Thus, the wet processing of NFC is a major challenge to find sustainable routes for NFC incorporation into the polymer matrix. In our case, freeze-drying almost doubled the required energy for production of the nanocellulose. Other methods of NFC polymer preparation often use organic solvents, which introduce other issues like toxicity and recovery of the expensive solvents [33]. Nanofillers often require much lower amounts to achieve similar properties than micro sized fillers so the case for lower NFC usage can make a difference [52]. To expand on this topic a full life cycle analysis is needed that takes into consideration other factors like PBS production costs, sustainable bio-based carbon content and recycling or waste processing.

### 3.2. Thermal Properties

**Figure 2:**
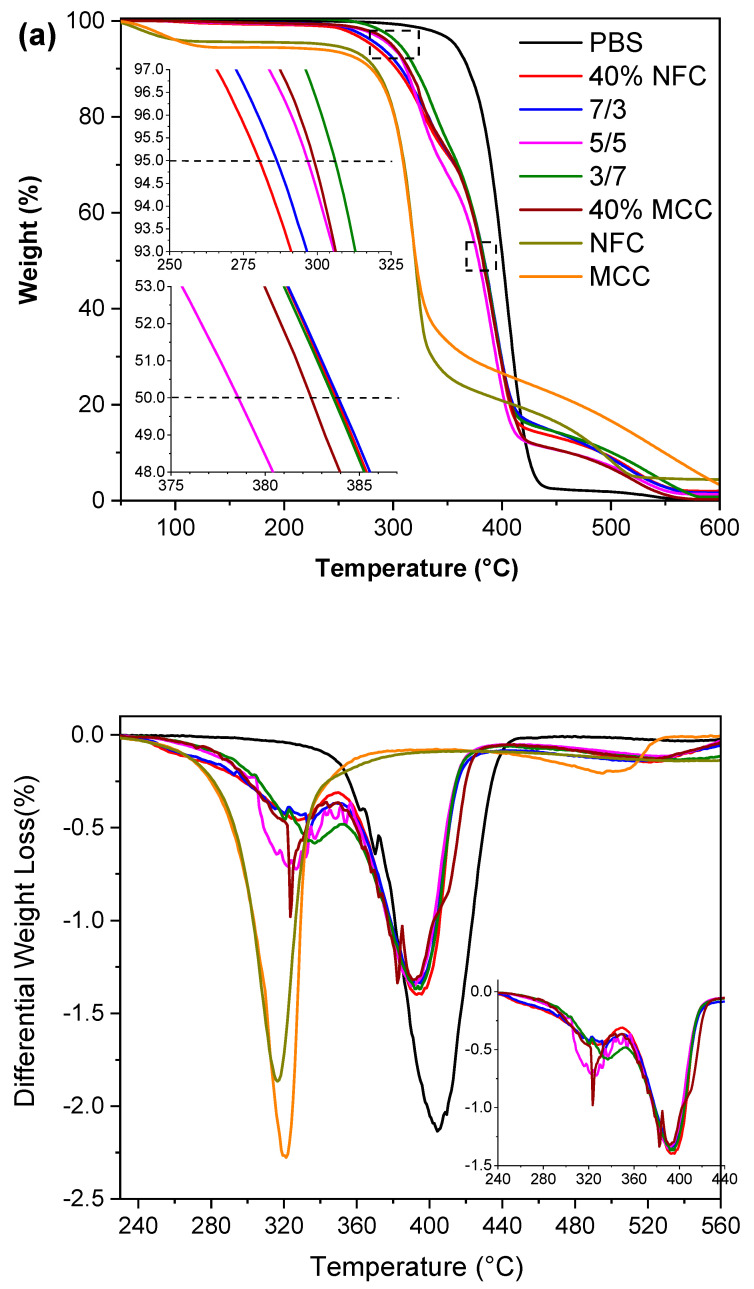
(**a**) Thermogravimetric analyses contours and (**b**) differential thermogravimetric contours of PBS, PBS/cellulose composites, NFC and MCC under air atmosphere.

The thermal stability along with degradation of the composite films was assessed using thermogravimetric analysis. Figure 2 shows the weight loss and the derivative weight loss curves of the pristine PBS, composites and NFC, MCC fillers. It can be observed from the differential weight loss curve, that PBS, NFC and MCC all have single-stage degradation curves while composites have two-stage degradations, from which the first can be attributed to cellulose filler and second to PBS matrix. NFC starts to degrade faster and has narrower degradation peak and it loses less of its overall weight compared to MCC, and this could be attributed to NFC structure that includes fibrils of varied nano sizes. PBS effectively shields cellulose filler increasing composite thermal degradation at 50% weight loss by 60 °C compared to MCC and NFC. Compared with pristine PBS, the thermal stability of composites was reduced. Weight loss curves indicate that cellulose has lower thermal stability and both NFC and MCC are similar and shows maximum weight loss at around 320 °C, while PBS has much higher temperature at 406 °C. The initial weight loss for the cellulose samples at around 100 °C has been attributed to the bonded water while the cellulose carbon skeleton pyrolysis starts at around 300 °C [53]. Table 3 summarizes TGA data from which the T_5%_ represents the thermal degradation temperature for 5% weight loss, T_50%_ represents the temperature of thermal degradation for 50% weight loss, T_max_ represents the extreme weight loss rate temperature.

Then the thermal stability of 40% NFC composite was 18 °C lower at T_5%_ compared to 40% MCC and composition 3/7 had the highest degradation temperature at 305 °C proving 25 °C and 7 °C increase respectively compared to single type cellulose specimens. For the values of T_50%_ and T_max_, all compositions showed similar values, except 40% MCC sample had 10 °C lower maximum weight loss temperature compared to other composites.

**Figure 3:**
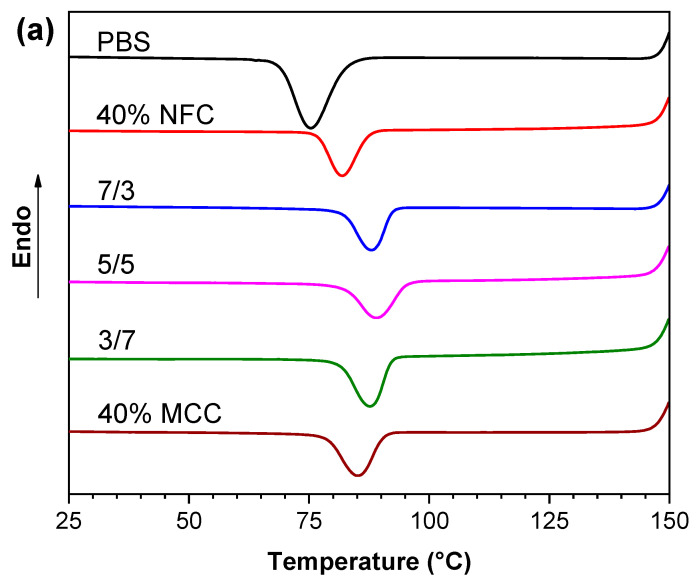

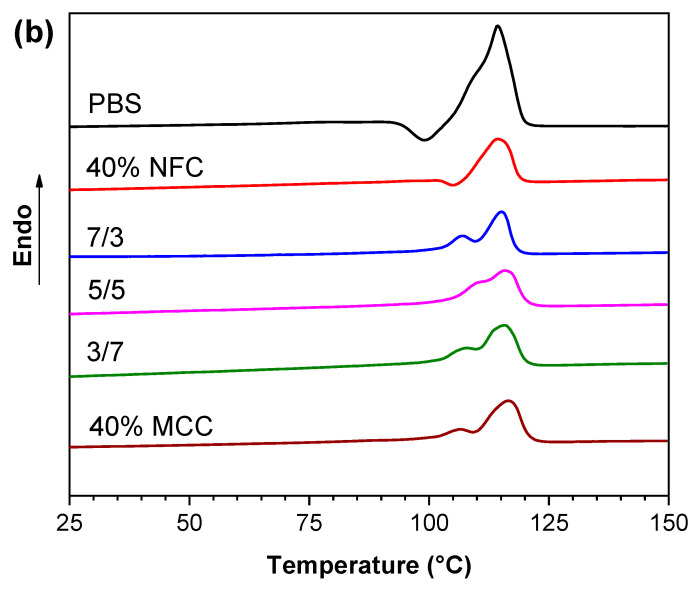
(**a**) Crystallization peaks of PBS and PBS/cellulose composites and (**b**) second heating melting peaks of PBS and PBS/cellulose composites under the nitrogen atmosphere.

DSC experiments were used to analyze the thermal and crystallization properties of PBS/cellulose composites. Table 3 displays the different thermal properties summary (e.g., crystallization temperature (T_c_), melting temperature (T_m_), crystallization enthalpy (H_c_), melting enthalpy (H_m_)), while the crystallization and melting curves are shown in Figure 3. Pristine PBS exhibited a sharp crystallization peak and crystallization temperatures (T_c_) at 75.3 °C. The addition of NFC shifted crystallization temperature to 81.9 °C, while the addition of MCC continued to enhance the crystallization temperature to 85.2 °C, but for NFC/MCC filler compositions, even higher values were observed of which 5/5 composition crystallization peak was at 89.0 °C. The crystallinity degree χ decreases with the addition of cellulose fillers from 35.5% for 40.0% MCC down to 29.1% for 40.0% NFC and NFC/MCC follow the same trend with higher NFC content, resulting in lower overall material’s crystallinity. It has been reported that cellulose fillers structure impacts the crystallization process, while agglomeration of NFC filler lowers crystallinity [54]. Thus, the degree of crystallization χ increases by higher MCC content, indicating heterogeneous nucleation of the PBS matrix [55], and the observed different values of crystallinity for 40% NFC and 40% MCC composites indicates strong dependence on the structure of cellulose filler. The observed curves indicate the polymer crystal nucleation by both fillers and pronounced trans-crystallization that can be observed as splitting in melting peak. For PBS/MCC composite this is observed as a spilt peak or second smaller peak [56]. The melting temperature of the composites is elevated compared to PBS and shows narrower peaks, which might be the result of trans-crystallization, as a result offset to higher temperatures is observed [57].

### 3.3. Thermomechanical and Tensile Properties

**Figure 4:**
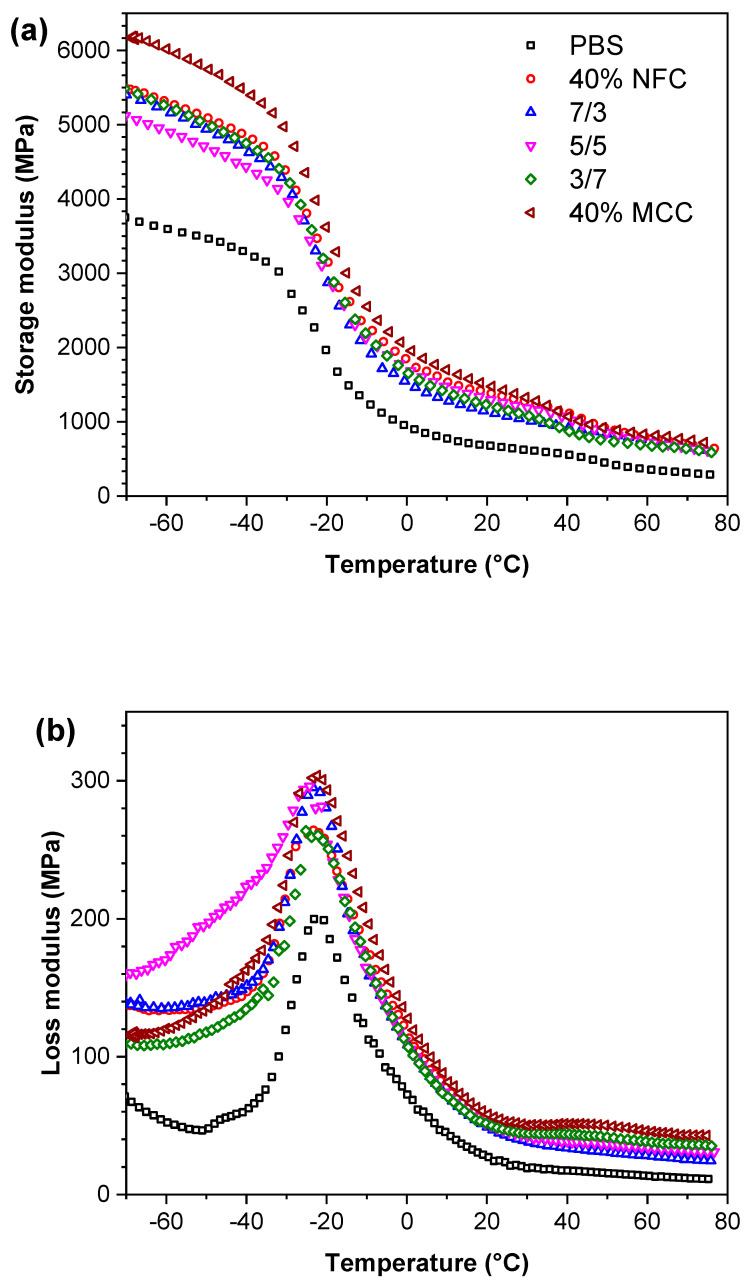

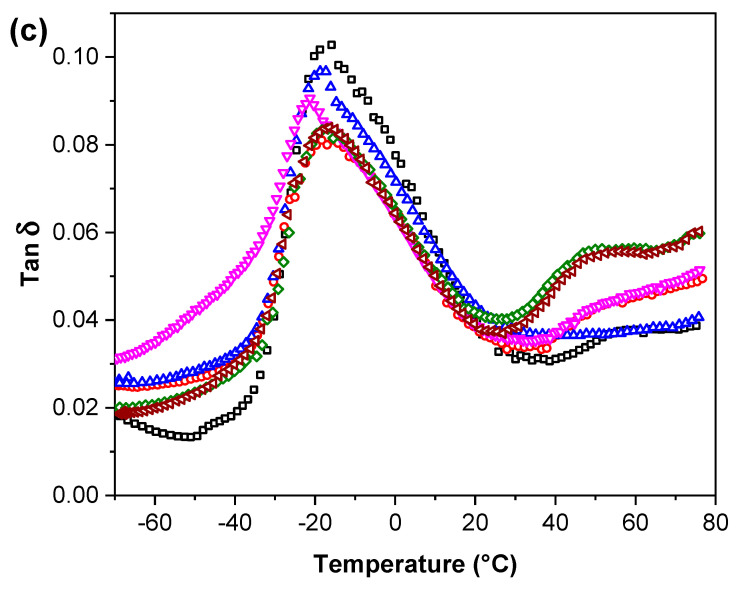
(**a**) The storage modulus E′; (**b**) the loss modulus E″ and (**c**) loss tangent tan δ curves of PBS and PBS/cellulose composites.

The thermo-mechanical properties of the PBS composites were studied with DMA. As evident from Figure 4a, the storage modulus of the prepared composites increases significantly with the addition of cellulose fillers to PBS matrix. The storage modulus was increased from 66% up to 119% at 20 °C with the lowest result for 7/3 composition and highest for 40% MCC, which showed the best enhancement in the whole temperature range compared to PBS. Similar trends were obtained for the loss modulus peaks (Figure 4b), which indicated the increase in the composite dampening properties, which saw an increase from 75% up to 100% at 20 °C, with 40% MCC having the highest observed value and 7/3 the lowest compared to PBS. However, before the glass transition temperature, composition 5/5 showed a significantly higher loss modulus than the rest of the composites. The storage and the loss modulus saw an increase in the entire array of temperature, which indicates a good filler dispersion and existing reinforcement network through the whole composite and good load-bearing properties [58]. The loss modulus increase can be explained by the particle–particle slippage, in this case, the cellulose filler and polymer PBS matrix that dissipated more heat than pure PBS [59]. The glass transition values as seen in tan δ peak (Figure 4c) ranges from −21 to −16 °C and are relatively unchanged for the composites compared to PBS value −16 °C. The absolute values of tan δ peak that characterize the energy dissipation were slightly decreased resulting in lower energy requirement for viscoelastic deformation of the composites, indicating weak interactions between PBS matrix and NFC/MCC fillers [60]. Thus, the storage and the loss modulus increase could be explained by the rigid MCC and NFC fibers proving the enhancement and reinforcement network.

**Figure 5:**
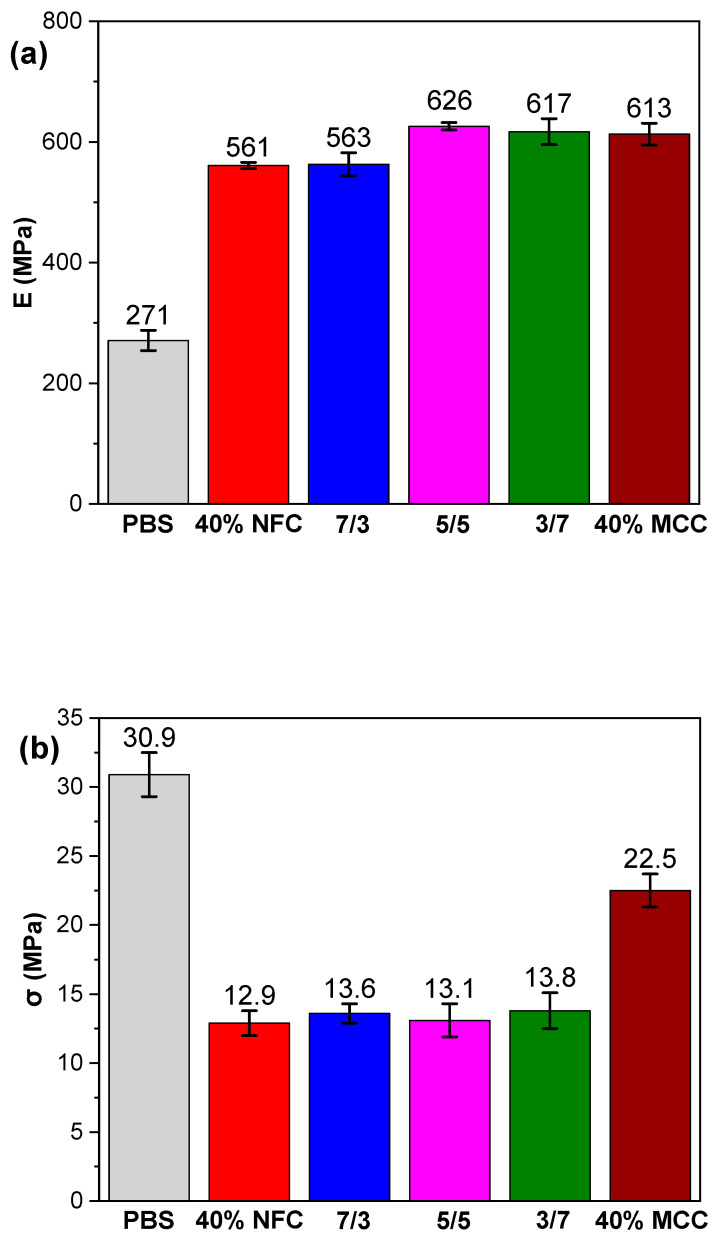

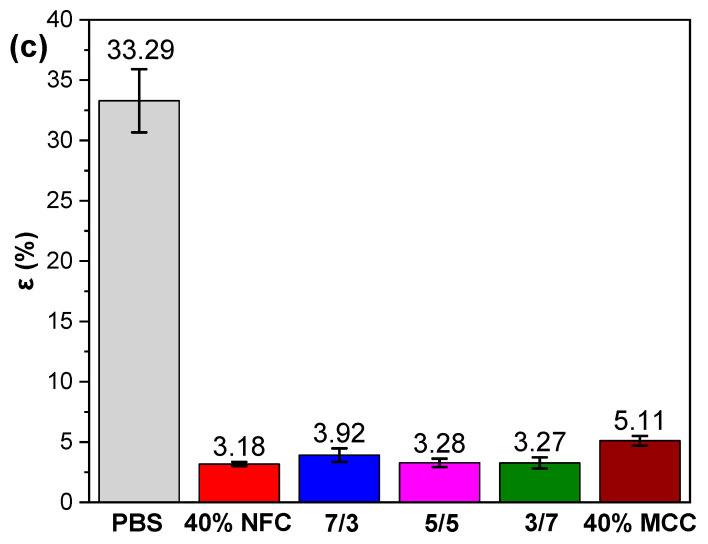
Tensile strength results of PBS and PBS/cellulose composites: (**a**) Elastic modulus E; (**b**) tensile strength σ and (**c**) strain ε.

Figure 5 shows mechanical properties for PBS/cellulose composites tested in tension mode and stress-strain curves are shown in Supplementary Figure S1. Addition of the cellulose fillers to PBS matrix causes an increase from 271 MPa to 561 MPa (107% increase) for 40% NFC with the highest value of 626 MPa (131% increase) for 5/5 in Young’s modulus, and this could be attributed to the inherited high modulus value of cellulose. The difference between filler crystallinity with similar composition has been shown to impact Young’s modulus and could explain the higher values achieved with compositions that have higher MCC content [61]. A synergic effect of the fillers has been observed for 5/5 composition, while 40% NFC and 7/3 compositions showed the lowest Young’s modulus. A decrease has been observed for tensile strength from 30.9 MPa for PBS down to 22.5 MPa for 40% MCC and lowest value 12.9 MPa for 40% NFC, with is a similar situation for the elongation values as 40% MCC has highest observed at 5.11% and 40% NFC lowest 3.18%. The decrease in tensile strength can directly be attributed to weak interactions between filler and matrix [61]. This results in stress concentrations that lead to brittle points in composite’s structure that reduce tensile strength and elongation values [62,63]. From hybrid compositions, the highest tensile strength was observed for 3/7. Thus, it can be observed that NFC shows poorer mixing with PBS matrix compared to MCC and this could be attributed to different structures, surface area and crystallinity of cellulose, which affects the amount of available OH groups and increase chemically bound water [64]. The ductility decrease has been explained by the increased volume of cellulose with high loading that enlarges the contact surface, which results in restricted polymer chain movements [65]. That is commonly observed as a sharp decline in elongation and has been reported for PBS/plant fiber compositions [43,66] and other polymers like polypropylene/wood composite systems [65,67].

### 3.4. Structure, Morphology and Surface Properties

**Figure 6:**
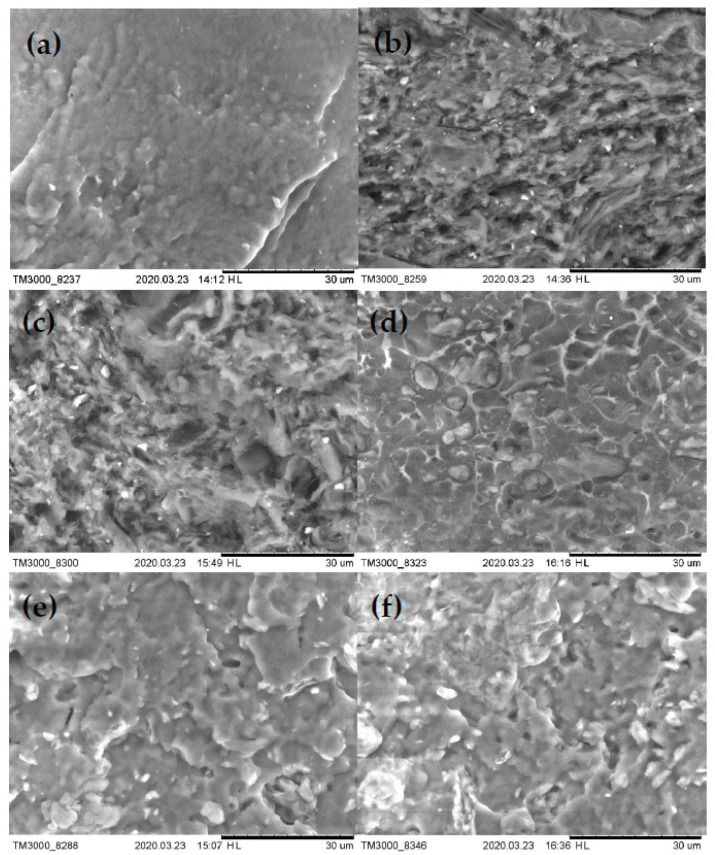
Scanning electron microscopy micrographs: (**a**) PBS; (**b**) 40% NFC; (**c**) 7/3; (**d**) 5/5; (**e**) 3/7 and (**f**) 40% MCC.

The SEM images of the fracture surfaces of pristine PBS and PBS/cellulose composites are shown in Figure 6 and in Supplementary Figure S2. SEM photographs indicate that the PBS matrix coverage of MCC and NFC differ significantly. The 40% NFC composition has voids and a very rough fracture surface that includes micropores. Addition of MCC to NFC resulted in a 7/3 hybrid that has a reduced number of voids and improved dispersion of filler, while surface roughness persists. It can be observed that the 5/5 hybrid composite has much fewer of the previously described defects, and they have become local, but the basic structure of surface roughness is more even and smoother. The 40% MCC and 3/7 composites have very similar fracture surfaces. There are no large voids compared to 40% NFC composition, the surface structure is smooth and has no visible micropores. The blend filler is evenly spread through matrix and there are no visible large agglomerates. Still, even in these two compositions, small voids can be observed and some smaller agglomerations with poorly covered cellulose can be observed. Tensile strength values achieved by 7/3 composition show that, even with very poor dispersion and compatibility, NFC can achieve acceptable results. Thus, application of for PBS/NFC blends should involve modification or grafting methods to achieve superior properties possessed by NFC.

**Figure 7:**
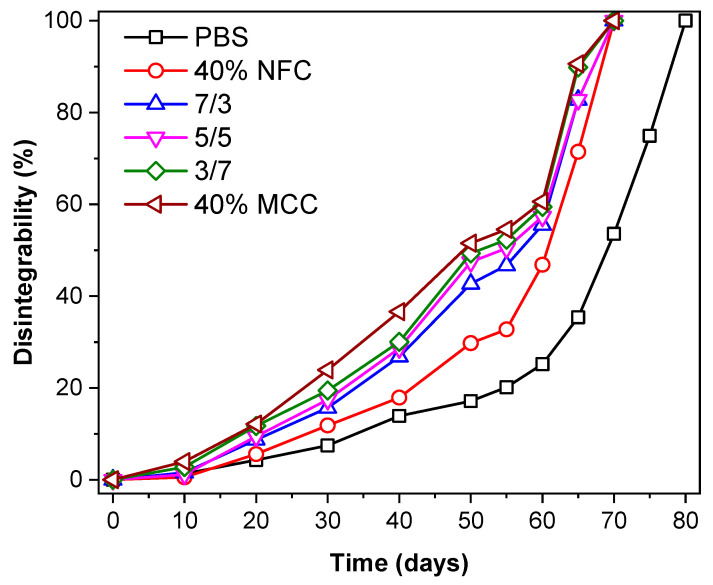
Percentage weight loss of PBS and PBS/cellulose composites in soil burial test conducted in composting conditions.

**Figure 8:**
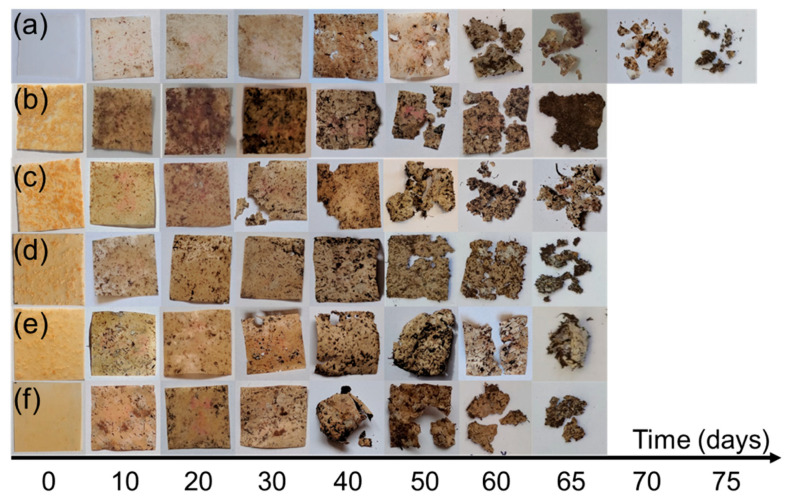
Photos of (**a**) PBS; (**b**) 40% NFC; (**c**) 7/3; (**d**) 5/5; (**e**) 3/7 and (**f**) 40% MCC films during biodegradation studies in soil burial test conducted in composting conditions.

The percentage weight loss in composting conditions for PBS and PBS/cellulose composites is presented in Figure 7. While visual specimen degradation can be observed in Figure 8, for the pristine PBS, it takes around 75 to 80 days to become almost indistinguishable from the soil. As it can be seen in the case of MCC and NFC filled compositions, it takes 10 days less for them to degrade, resulting in a total time of around 65 to 70 days. We also observed that PBS/cellulose composites lose their ductility much faster and become brittle and can easily crumble with slight pressure, while pristine PBS retained mechanical toughness longer. The composition with MCC showed slightly enhanced degradation compared to NFC ones. It has been reported before that cellulose-based filler enhances the degradation of PBS in composting conditions and the weight percentage of filler determines the impact on the degradation time [42]. A hydrolysis mechanism is the first stage of PBS degradation and requires enzymatic activity in soil and water, while degradation products are processed by bacteria and fungi [45,68]. It has been shown that the amount of water, temperature and the composition of soil influences degradation time significantly [69,70]. Other factors like crystallinity also impact the speed of degradation and generally amorphous regions of polymers degrade faster than crystalline ones [45]. So, compositions with MCC indicated faster degradation than ones with more NFC, which could be explained by better filler distribution in the polymer matrix, as can be observed in SEM images. Thus, better compatibility and distribution of MCC in the blend allows composting properties to be further enhanced.

Contact angle measurements were used in addition to biodegradation studies to determine surface wettability. Table 4 summarizes the measured contact angle (Θ) values with standard deviation of distilled water for pristine PBS and PBS/cellulose composite films, surface free energy (SFE) calculated with the Owen, Wendt, Rabel and Kaelble (OWRK) method (total SFE of solids abbreviated as tot composed of dispersive component r_s_^d^ and polar component r_s_^p^) and Zisman method (γ_cr_ critical SFE of solids) using 3 different solutions (distilled water, glycerol and diiodomethane). The measured contact angles and Zisman plots are shown in Supplementary Figures S3 and S4, respectively. The contact angle values are lower for PBS/cellulose compositions compared to PBS, which means that the surface of the composites has improved wettability. Hybrid systems showed lower contact angle value compared single cellulose filler, with the lowest result for 1/1 composition. Zisman and OWRK methods showed that SFE decreased with the addition of cellulose, but the polar component was increased compared to PBS. Hybrid systems showed lowest SFE, while their polar component was higher than PBS and single filler systems. All compositions show that polymer covers cellulose fillers effectively, while lower contact angle values and higher polar component indicate improved wettability, which plays a role in the biodegradation process. As observed in the degradation experiment in composting conditions (Figure 8), PBS effectively covers cellulose filler and protects the surface from water and microorganisms until the polymer layer is degraded.

The insights presented in this research have been combined with literature analysis to present a discussion for selection of the filler. The MCC application in powder form makes it suitable for melt processing, showing good filler dispersion in varied concentration even up to 70 wt % [50]. Thus, the micro sized cellulose fillers offer economic, environmental benefits and are suitable for conventional melt processing methods. This results in systems that can be highly loaded with microcellulose and are renewable with high bio-based carbon content, which is not always the case for bio-based polymers that are often partly oil-based. NFC is generally used in low concentrations up to 10 wt % due to agglomeration issues that may arise [33]. Wet processing is considered standard for NFC and film casting or freeze-drying is done by dispersing in water soluble polymers to preserve nanostructure [34]. Some studies suggest that nanostructure of NFC can be preserved after freeze drying [35]. Crystalline cellulose fillers are often easier to disperse in polymer matrix, while fibrils tend to agglomerate. Very high mechanical properties and the high aspect ratio combined with nano size means that only small amounts of NFC are needed for reinforcement of the polymer matrix, and higher concentration presented diminishing affect returns or even decreased performance.

## 4. Conclusions

PBS polymer matrix can effectively shield the cellulose fillers from thermal degradation. As a result, the lower mass-loss rate was observed, and degradation occurred over a wider and upper-temperature range. The composites crystallization temperature was also increased, indicating heterogeneous nucleation of PBS stimulated by cellulose fillers. The crystallinity saw a decrease due to cellulose limiting PBS chain mobility. The composites storage modulus was found to increase significantly in the measured temperature range, confirming an effective filler reinforcement network. The tensile test showed that Young’s modulus improved about two fold, whereas tensile strength decreased along with elongation values. Surface wetting with water showed a slight decrease in observed contact angle values and increased polar component of the surface free energy. This was confirmed with weight loss measurements in composting conditions, where composites disintegrated 10 days faster than the pristine PBS sample. Thus, PBS/cellulose films would start to biodegrade faster, but still have a good resistance during the product lifetime. The SEM images of the fracture surfaces displayed that MCC and NFC pose very different compatibility with PBS, which coincided with results observed in the mechanical and dynamical mechanical analysis. A life cycle inventory for the fillers revealed an almost two-fold increase in energy consumption for NFC compared to MCC. We concluded that NFC having a higher surface area resulted in stronger hydrogen bonding, which leads to the formation of large agglomerates and uneven filler distribution. Freeze-drying is not an optimal processing method for unmodified NFC and wet processing is advisable. So, in the case of unmodified cellulose filler application for PBS composites, MCC is more suitable and economically viable.

## Figures and Tables

**Table 1:** The prepared poly (butylene succinate) (PBS)/cellulose compositions.

Sample	PBS wt %	NFC wt %	MCC wt %
PBS	100	0	0
40% NFC	60	40	0
7/3	60	28	12
5/5	60	20	20
3/7	60	12	28
40% MCC	60	0	40

**Table 2:** Materials and energy flows for the laboratory manufacture of the MCC and NFC.

Input	Unit	Quantity per Declared Unit (1 kg of Composite)	
		MCC	NFC
*Materials*			
Softwood kraft pulp	kg	1.010	1.010
Hydrochloric acid 0.05 wt %	L	20	20
Distilled water	L	2	12
*Electric energy*			
Drying	kWh	224	224
Jar milling	kWh	210	210
Freeze-drying	kWh	-	417.6
Total energy	kWh	434	851.6
*Emissions*			
Dust	kg	0.001	0.001
Solid waste	kg	0.009	0.009
Acidic water	L	20	20

**Table 3:** Thermal properties of PBS/cellulose composites.

Sample	T_m_ (°C)	T_c_ (°C)	H_m_ (J/g)	H_c_ (J/g)	χ (%)	T_5%_ (°C)	T_50%_ (°C)	T_max_ (°C)
PBS	114.2	75.3	75.1	72.9	36.5	356	400	406
40% NFC	114.2	81.9	31.8	35.0	29.1	280	384	392
7/3	115.1	88.1	34.5	36.8	30.6	286	384	392
5/5	115.8	89.0	37.7	37.2	31.0	296	379	392
3/7	115.8	87.6	39.0	38.2	31.8	305	383	395
40% MCC	116.5	85.2	38.8	42.6	35.5	298	382	383

**Table 4:** Surface properties of PBS and PBS/cellulose composites.

Sample	Distilled Water		OWRK			Zisman
	Θ (deg)	SD	Tot (mN/m)	r_s_^d^ (mN/m)	r_s_^p^ (mN/m)	γ_cr_ (mN/m)
PBS	77.1	1.4	43.7	40.4	3.3	43.4
40% NFC	76.4	1.0	42.3	39.0	3.3	41.9
7/3	75.4	0.7	41.7	38.4	3.3	41.3
5/5	73.5	1.1	41.5	37.5	4.0	39.5
3/7	75.0	0.8	42.9	39.1	3.8	41.6
40% MCC	76.9	1.7	42.5	39.2	3.3	42.6

## Supplementary Materials

The following are available online at https://www.mdpi.com/2073-4360/12/7/1472/s1, Figure S1: Example of the characteristic tensile curves of PBS/cellulose composites, Figure S2: Scanning electron microscopy micrographs: (a) PBS; (b) 40% NFC; (c) 7/3; (d) 5/5; (e) 3/7 and (f) 40% MCC, Figure S3: The measured contact angles: (a) 40% MCC; (b) 3/7; (c) 5/5; (d) 7/3; (e) 40% NFC and (f) PBS, Figure S4: Zisman plots: (a) 40% MCC; (b) 3/7; (c) 5/5; (d) 7/3; (e) 40% NFC and (f) PBS.
