# Supplementary material for: Bio-Based Poly(butylene succinate)/Microcrystalline Cellulose/Nanofibrillated Cellulose-Based Sustainable Polymer Composites: Thermo-Mechanical and Biodegradation Studies
Source: Polymers (Basel). 2020 Jun 30;12(7):1472. doi: 10.3390/polym12071472 (PMC7408463; doi:10.3390/polym12071472)
Supplement: Supplementary file 1 [file polymers-12-01472-s001.pdf]

Supplementary Materials

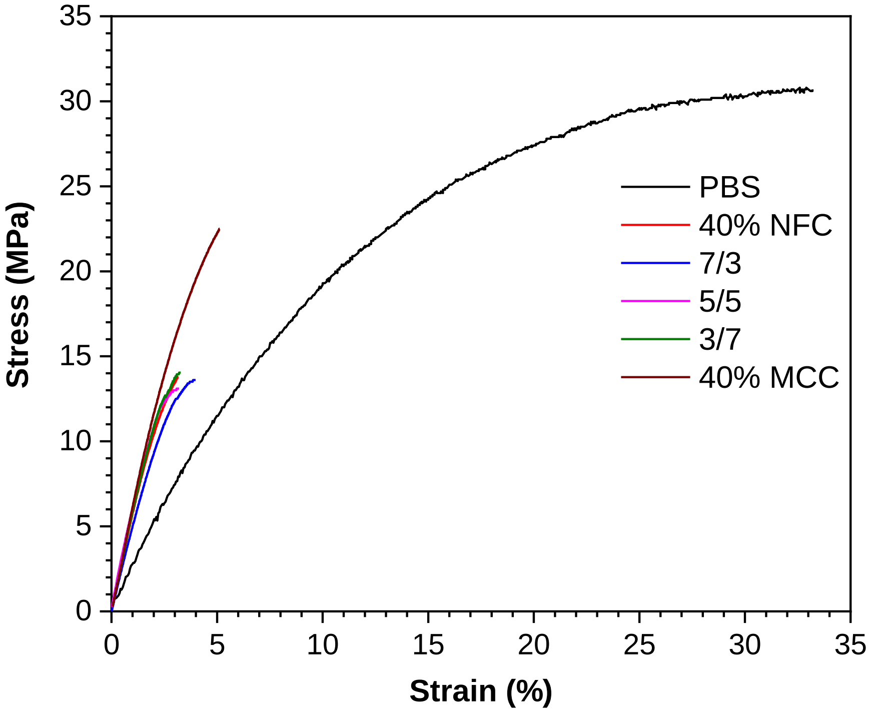

Figure S1. Example of the characteristic tensile curves of PBS/cellulose composites.

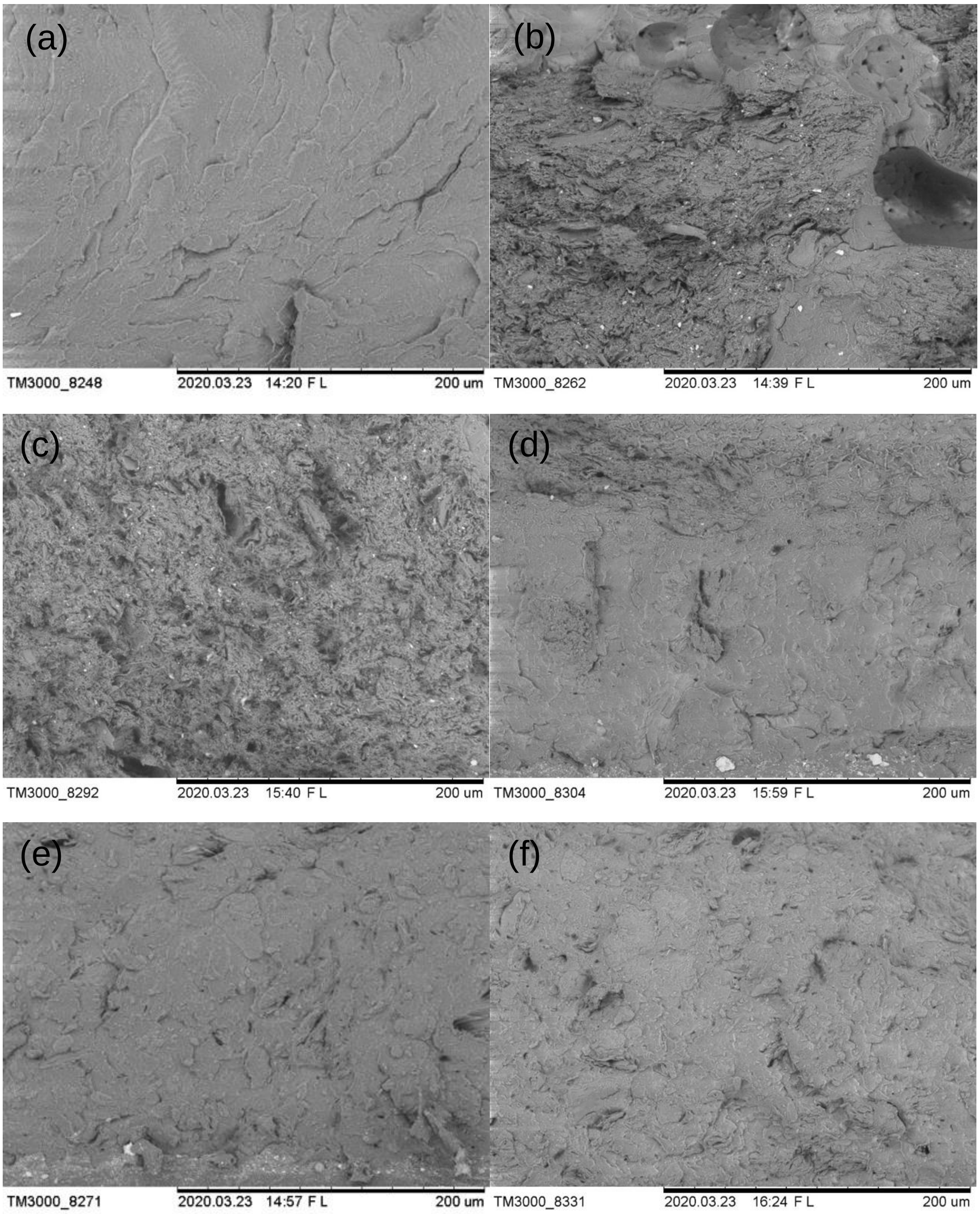

Figure S2. Scanning electron microscopy micrographs: **(a)** PBS; **(b)** 40% NFC; **(c)** 7/3; **(d)** 5/5; **(e)** 3/7 and **(f)** 40% MCC.

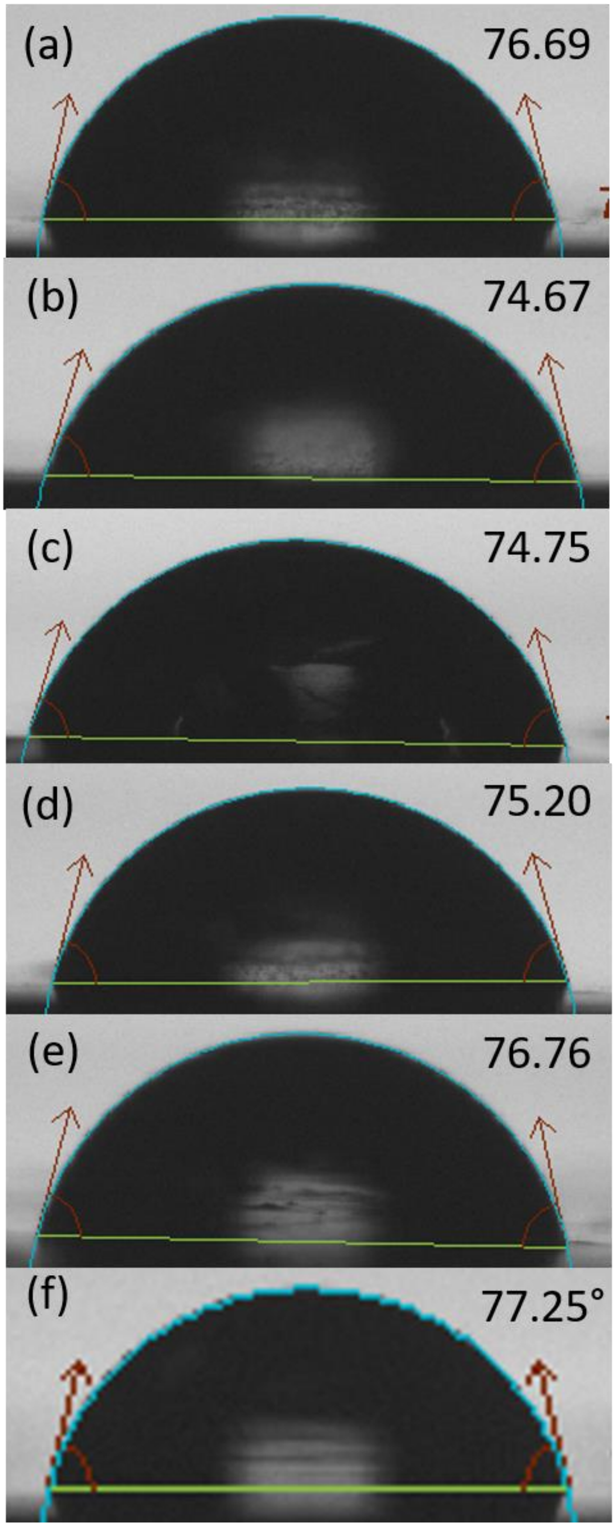

Figure S3. The measured contact angles: **(a)** 40% MCC; **(b)** 3/7; **(c)** 5/5; **(d)** 7/3; **(e)** 40% NFC and **(f)** PBS.

(a)

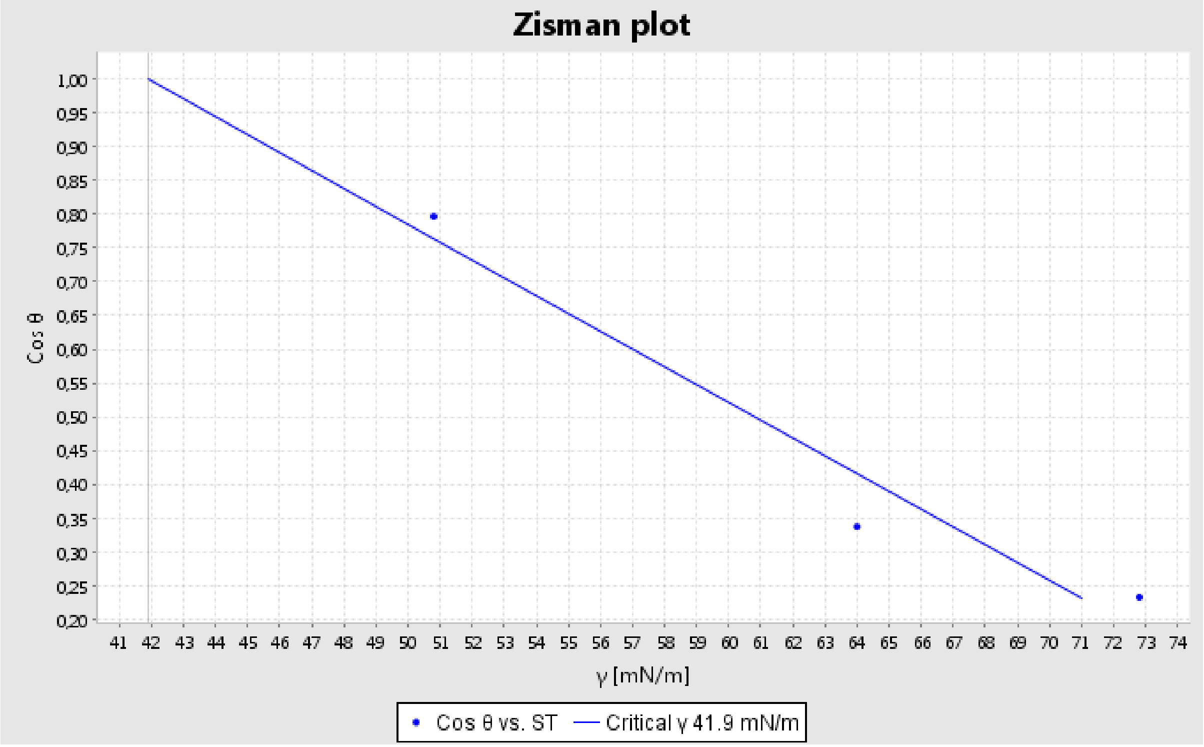

Zisman plot
Cos θ
γ [mN/m]
Cos θ vs. ST
Critical γ 41.9 mN/m

(b)

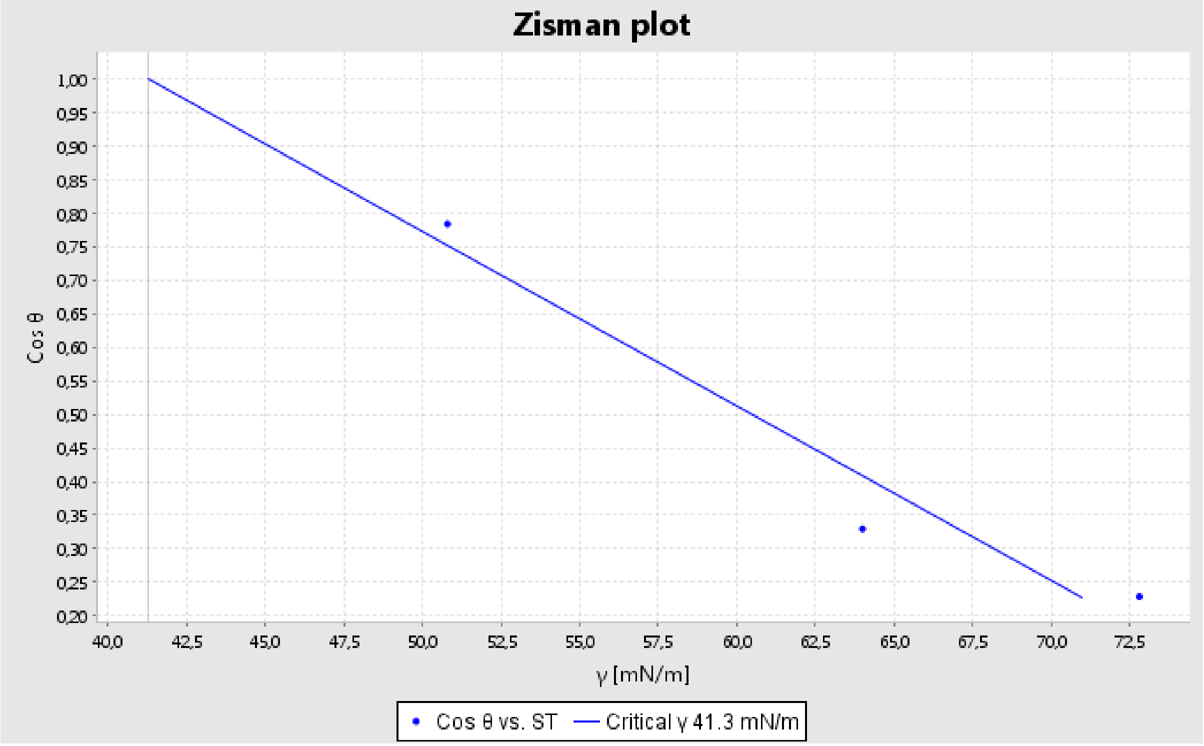

Zisman plot
Cos θ
γ [mN/m]
Cos θ vs. ST
Critical γ 41.3 mN/m

(c)

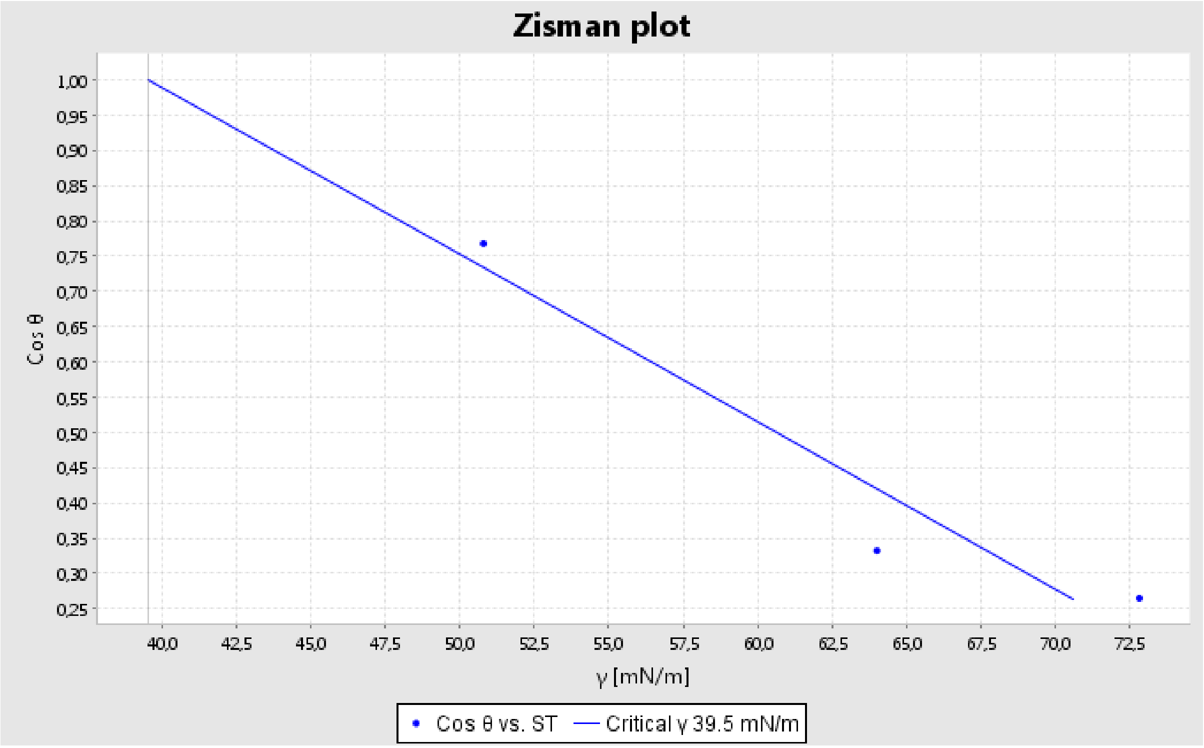
Zisman plot
Cos θ
1,00
0,95
0,90
0,85
0,80
0,75
0,70
0,65
0,60
0,55
0,50
0,45
0,40
0,35
0,30
0,25
40,0
42,5
45,0
47,5
50,0
52,5
55,0
57,5
60,0
62,5
65,0
67,5
70,0
72,5
γ [mN/m]
Cos θ vs. ST
Critical γ 39.5 mN/m

(d)

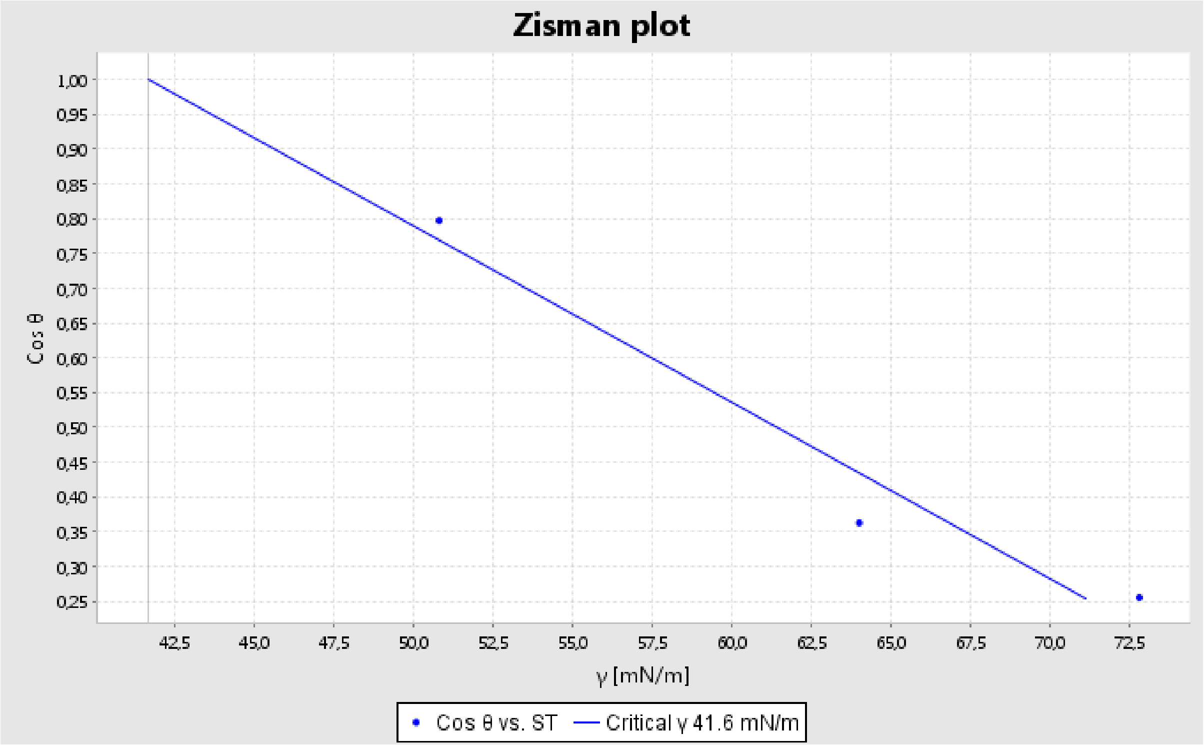
Zisman plot
Cos θ
1,00
0,95
0,90
0,85
0,80
0,75
0,70
0,65
0,60
0,55
0,50
0,45
0,40
0,35
0,30
0,25
42,5
45,0
47,5
50,0
52,5
55,0
57,5
60,0
62,5
65,0
67,5
70,0
72,5
γ [mN/m]
Cos θ vs. ST
Critical γ 41.6 mN/m

(e)

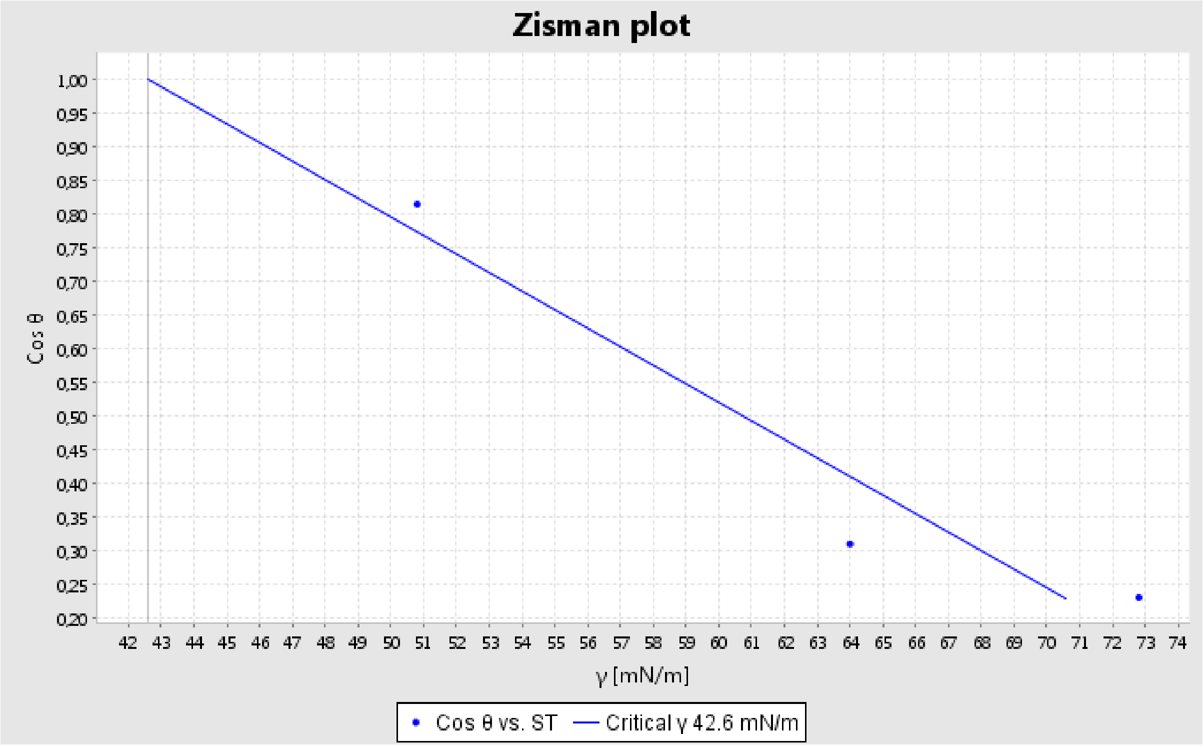

(f)

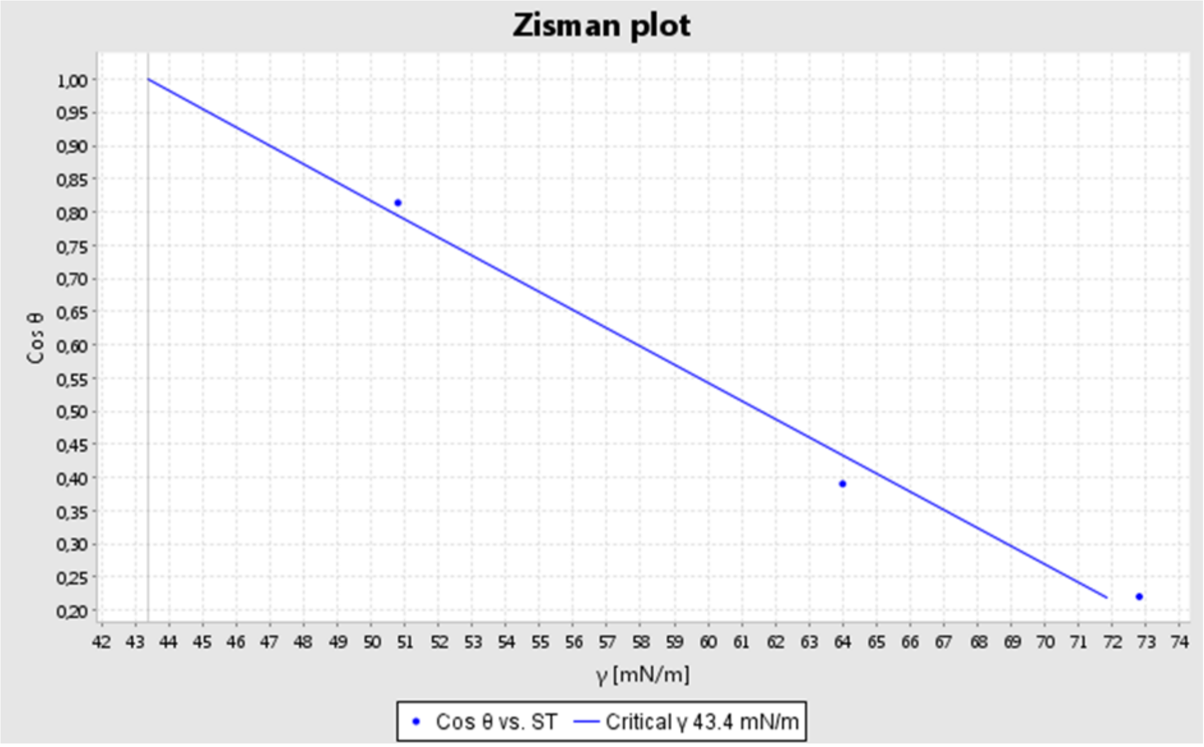

Figure S4. Zisman plots: **(a)** 40% MCC; **(b)** 3/7; **(c)** 5/5; **(d)** 7/3; **(e)** 40% NFC and **(f)** PBS.
